# Defective base excision repair in the response to DNA damaging agents in triple negative breast cancer

**DOI:** 10.1371/journal.pone.0223725

**Published:** 2019-10-09

**Authors:** Kevin J. Lee, Cortt G. Piett, Joel F. Andrews, Elise Mann, Zachary D. Nagel, Natalie R. Gassman

**Affiliations:** 1 Mitchell Cancer Institute, University of South Alabama, Mobile, AL, United States of America; 2 University of South Alabama College of Medicine, Mobile, AL, United States of America; 3 Harvard T.H. Chan School of Public Health, Harvard University, Boston, MA, United States of America; Tel-Aviv University, ISRAEL

## Abstract

DNA repair defects have been increasingly focused on as therapeutic targets. In hormone-positive breast cancer, XRCC1-deficient tumors have been identified and proposed as targets for combination therapies that damage DNA and inhibit DNA repair pathways. XRCC1 is a scaffold protein that functions in base excision repair (BER) by mediating essential interactions between DNA glycosylases, AP endonuclease, poly(ADP-ribose) polymerase 1, DNA polymerase β (POL β), and DNA ligases. Loss of XRCC1 confers BER defects and hypersensitivity to DNA damaging agents. BER defects have not been evaluated in triple negative breast cancers (TNBC), for which new therapeutic targets and therapies are needed. To evaluate the potential of XRCC1 as an indicator of BER defects in TNBC, we examined XRCC1 expression in the TCGA database and its expression and localization in TNBC cell lines. The TCGA database revealed high XRCC1 expression in TNBC tumors and TNBC cell lines show variable, but mostly high expression of XRCC1. XRCC1 localized outside of the nucleus in some TNBC cell lines, altering their ability to repair base lesions and single-strand breaks. Subcellular localization of POL β also varied and did not correlate with XRCC1 localization. Basal levels of DNA damage correlated with observed changes in XRCC1 expression, localization, and measure repair capacity. The results confirmed that XRCC1 expression changes indicate DNA repair capacity changes but emphasize that basal DNA damage levels along with protein localization are better indicators of DNA repair defects. Given the observed over-expression of XRCC1 in TNBC preclinical models and tumors, XRCC1 expression levels should be assessed when evaluating treatment responses of TNBC preclinical model cells.

## Introduction

Defects in DNA damage response and repair are driving factors in carcinogenesis and key determinants for chemotherapeutic response. Breast cancers may display defects in DNA repair such as mutations in key DNA damage response and repair proteins such as breast cancer-susceptibility gene (*BRCA1/2*) and tumor suppressor protein p53 (*TP53*), and altered expression levels of DNA repair proteins thymine-DNA glycosylase (TDG) and poly(ADP-ribose) polymerase 1 (PARP1) [1–3]. Exploiting DNA repair defects present in cancer cells but absent in normal cells improves therapeutic responses, as demonstrated by the use of PARP-inhibitors (PARPi) in cancers that have BRCA1/2 deficiencies. However, characterization of DNA repair pathways in preclinical models is often lacking. DNA repair defects modulate the efficacy of therapeutic agents, and their characterization in preclinical models and patients is essential for evaluating therapeutic options.

In breast cancer, DNA repair defects often extend beyond homologous recombination defects. Observed changes in the expression of base excision repair (BER) proteins such as TDG and DNA polymerase beta (POL β) demonstrate other DNA repair pathways contribute to genomic instability and may alter therapeutic responses [2, 3]. Single nucleotide polymorphisms in BER genes such as POL β, PARP1, and X-ray cross complementing protein 1 (XRCC1) are associated with increased risk of developing breast cancer [4–7]. However, the roles of BER proteins in breast cancer development and treatment are poorly understood.

XRCC1 is a critical scaffold protein in BER that facilitates critical protein-protein interactions at the site of DNA damage, mediating associations among DNA glycosylases, AP endonuclease 1 (APE1), PARP1, POL β, and DNA ligase III (LIG3). XRCC1’s facilitation of protein-protein interactions also provides for overlap in functions between BER and single-strand break repair (SSBR) pathways [8, 9]. XRCC1 also participates in double-strand break (DSB) repair through its interaction with PARP1 in the error-prone alternative nonhomologous end joining (NHEJ) [10–12], and with DNA LIG3 in nucleotide excision repair (NER) [13].

XRCC1 is ubiquitously expressed in normal tissues, but low levels of XRCC1 cause impaired BER in terminally differentiated muscle cells and neurons [14, 15]. Variations in XRCC1 expression are observed in breast cancer patient samples [16–18], with low XRCC1 expression proposed as a target for PARPi treatment [16–19]. Defects in BER related to low XRCC1 expression may sensitize breast cancer cells to PARPi, similar to homologous recombination and BRCA1/2 defects [16, 18, 20, 21]. However, the frequency of XRCC1-deficiency in breast cancer is not known, and the susceptibility to PARPi across breast cancer subtypes is undefined.

Defects in BER, including XRCC1 expression level changes, have not been fully explored in triple negative breast cancers (TNBC). TNBC is associated with rapid growth, high metastatic potential, and poor overall 5-year prognosis. TNBC primary tumors have a high level of genomic instability and a high prevalence of DNA repair defects, though not always associated with mutations in the *BRCA1/2* genes [1, 16–18, 22]. Further, TNBC lack the estrogen receptor (ER), progesterone receptor (PR), and human epidermal growth factor receptor 2 (HER2), which are the molecular targets for current breast cancer therapies. The lack of these molecular targets in TNBC limits treatment options for TNBC to surgery and DNA damaging radiotherapy or chemotherapy.

Therefore, DNA repair defects in TNBC can play a critical role in dictating the response to therapy. With changes in XRCC1 expression observed in some breast cancers, we hypothesized that XRCC1 expression level changes in TNBC could indicate BER defects which could be targeted to provide new treatment strategies for this aggressive breast cancer subtype.

## Materials and methods

### The Cancer Genome Atlas analysis

The Cancer Genome Atlas (TCGA) analysis was performed with an internet resource (UALCAN: http://ualcan.path.uab.edu) as previously reported by Chandrashekar et al. [23] and plotted as a Box and Whisker to show data distribution.

### Cell culture

MDA-MB-157 (MDA-157), MDA-MB-231(MDA-231) and MDA-MB-468 (MDA-468), MCF10A, and HCC1806 cells were purchased from the American Type Culture Collection (ATCC #’s HTB-24, HTB-26, HTB-132, CRL-10317, and CRL-2335, respectively) within the last 12 months and passaged < 15 times for all experiments. Cells were tested biweekly during experiments for mycoplasma contamination using the Lonza MycoAlert (Lonza #LT07-318). HCC1806 cells were grown in RPMI 1640 Medium (Life Technologies #11875093) and supplemented with 10% Fetal Bovine Serum (FBS, Atlantic Biologicals Premium Select). MDA-157, MDA-231, MDA-468, and MCF10A cells were grown in DMEM High Glucose + GlutaMAX (Life Technologies #10566016) and supplemented with 1% sodium pyruvate (Life Technologies #11360070) and 10% FBS. Cells were maintained in a humidified 37°C incubator with 5% carbon dioxide.

### shRNA knockdown of XRCC1

Plasmid constructs for stable depletion of human XRCC1 mRNA were obtained from Sigma-Aldrich (MISSION shRNA). Two different shRNAs against XRCC1 were used: **XRCC1 shRNA 1**- (TRCN000011211): CCGGCGATACGTCACAGCCTTCAATCTCGAGATTGAAGGCTGTGACGTATCGTTTTT **XRCC1 shRNA 2**- (TRCN0000007913): CCGGCCAGTGCTCCAGGAAGATATACTCGAGTATATCTTCCTGGAGCACTGGTTTTT. pKLO.1 plasmid without insert was also purchased from Sigma-Aldrich and transfected in parallel with shRNAs. MDA-468 cells were plated at 40,000 cells per well into a 12-well dish. The next day cells were transfected with 0.5 μg plasmid DNA and JetPrime (Polyplus) at a 1:2 ratio of DNA to JetPrime. Cells were allowed to recover for 48 h following transfection; then stable cell lines were recovered after puromycin selection (0.5 μg/ml, Life Technologies). Single-cell clones were isolated and after immunoblot analysis, those demonstrating significant XRCC1 knockdown were characterized. No difference in XRCC1 expression or cell sensitivity was observed with the MDA-468 pLKO.1 control cell line compared to the MDA-468 parental.

### Immunoblot

Immunoblot was performed as described previously [24]. Cells were grown in 150 mm dishes and cultured to 70–80% confluence. Cells were rinsed with PBS, scraped, stored overnight at -80°C, then lysed. Lysates were separated on a 4–15% SDS Page gel (Biorad), and transferred to a nitrocellulose membrane. The membrane was then probed with antibodies diluted in 5% non-fat dry milk in Tris buffered saline (VWR #J640-4L) and 0.1% Tween20 (Fisher Scientific #BP337, TBS-T) and raised against PARP1 (Cell Signaling #9532) that was diluted 1:1000, XRCC1 (Fisher Scientific #MS434P1) that was diluted 1:500, p53 (Santa Cruz Biotechnology #sc-6243) that was diluted 1:1000, BRCA1 (Novus Biologicals #NBP1-45410) that was diluted 1:500, and glyceraldehyde 3-phosphate dehydrogenase (GAPDH) (Santa Cruz Biotechnology #sc-365062) that was diluted 1:1000. The blots were incubated with either horseradish peroxidase (HRP)-labeled secondary antibodies: goat anti-rabbit-HRP or goat anti-mouse-HRP (Cell Signaling Technology). HRP antibody target proteins were detected by incubating with WesternBright Sirius (Advansta).

All immunoblotting was conducted with three biological replicates. Where indicated, protein quantification was conducted with Image Lab Software (BioRad). Band intensity was normalized to loading controls and averaged over the three biological replicates.

### Subcellular fractionation

Nuclear and cytoplasmic fractions were prepared using the NE-PER Nuclear and Cytoplasmic Extraction reagents (Thermo Scientific #78835). Briefly cells were grown to 80% confluency. The cells were then placed on ice, washed in ice-cold PBS, and harvested with 0.25% Trypsin-EDTA (Thermo Fisher) and centrifuged at 500 x g for 5 min. The cell pellets were resuspended in 50 μl of ice-cold Cytoplasmic Extraction Reagent I (CER I). The MDA-157, MDA-231, and HCC1806 cells lines were vortexed at the highest setting for 15 sec to resuspend the cell pellet and placed on ice for 10 min. The MDA-468 cells were gently resuspended by flicking the tube and incubated on ice for 10 min. Then 2.25 μl of ice-cold Cytoplasmic Extraction Reagent II (CER II) was added to all tubes. The MDA-157, MDA-231, MDA-468, and HCC1806 cells lines were then vortexed at the highest setting for 5 sec and placed on ice for 1 min. The MDA-157, MDA-231, and HCC1806 cells lines were then vortexed again at the highest setting for 5 sec, while the MDA-468 remained on ice. The cells were then centrifuged at 16,000 x g for 5 min at 4˚C. Cytoplasmic fraction (supernatant) was then transferred to a clean, pre-chilled tube. Nuclear extraction was carried out according to the manufacturer’s protocol. Protein concentration was quantitated using the Bradford protein assay kit (BioRad). Equal amounts of cytoplasmic extract (~ 80 μg) and nuclear extract (5 μg) were loaded in each lane and separated on a 10% Criterion TGX Stain-Free Protein Gel (BioRad), then transferred onto a nitrocellulose membrane. Membranes were probed with the following primary antibodies: mouse anti-XRCC1 (clone 33-2-5) (Thermo Fisher, MA513412), mouse anti-β-actin (Thermo Fisher, AM4302), mouse anti-Lamin A/C (Cell Signaling Technology, 4777). The blots were incubated with either horseradish peroxidase (HRP)-labeled secondary antibodies: goat anti-rabbit-HRP or goat anti-mouse-HRP (Cell Signaling Technology). HRP antibody target proteins were detected by incubating with WesternBright Sirius.

### Immunofluorescence

Cells were plated in 8-well slides (Nunc Chambered Coverglass, ThermoFisher). Wells were coated with 250 μL EmbryoMax 0.1% Gelatin Solution (Millipore #ES-006-B) for 15–30 min at room temperature (RT ~ 23°C), EmbryoMax was removed and cells were added at 3 x 10^4^ cells per well and allowed to reach 70–80% confluence before fixation in 3.7% formaldehyde (Fisher Scientific #BP531) in PBS for 10 min at RT, then washed 3x in PBS. The cells were permeabilized using Permeabilization Buffer (Biotium #22016) for 10 min at RT, washed 3x in PBS, and blocked using 2% bovine serum albumin (BSA, Jackson ImmunoResearch #001-000-173) in PBS for 30 min at RT. Primary antibodies were diluted in 2% BSA in PBS and incubated for 1 h at RT. XRCC1 (abcam #ab1838) was diluted 1:100; POL β (abcam #ab26343) was diluted 1:200; 53BP1 (Novus #NB100-304) was diluted 1:750; γH2AX-647 (Millipore #05-636-AF647) was diluted 1:750; Poly ADP-Ribose Polymer (PAR) (abcam #ab14460) was diluted 1:200; p53 (Life Technologies #MA514516) was diluted 1:200; and PARP1 (Santa Cruz Biotechnology #sc53643 clone C2-10) was diluted 1:200. After primary incubation, cells were washed 3x in PBS and secondary antibodies were diluted 1:2000 in 2% BSA and incubated for 45 min at RT in the dark. Secondary antibodies were either goat anti-Mouse Alexa Fluor 546 (Invitrogen #A11003), goat anti-rabbit Alexa Fluor 546 (Invitrogen #A11010), goat anti-Mouse Alexa Fluor 488 (Invitrogen #A11001), or goat anti-Rabbit Alexa Fluor 488 (Invitrogen #A11008). One drop of NucBlue Fixed Cell Reagent (ThermoFisher #R37606) was added to each chamber and chambers were incubated for 15 min at RT. Cells were washed 2x in PBS and imaged immediately for analysis or stored at 4°C overnight. Fluorescence images were acquired using a Nikon A1r scanning confocal microscope with a Plan-Apochromat 20x/0.75 objective. For quantification, the region of interest (ROI) generator was used to automatically detect nuclei in the DAPI channel, and the mean intensity in the channel of interest was exported for analysis. For XRCC1 and POL β nucleus to cytoplasmic ratio (N/C), nuclei were defined automatically and the whole cell was drawn using Bezier function. The nucleus was then subtracted from the whole cell intensity to determine cytoplasmic intensity described previously [25, 26].

### DNA damage analysis utilizing RADD

Cells were plated in 8-well chambered coverglass, fixed, and permeabilized using the same procedure as for immunofluorescence. The Repair Assisted Damage Detection (RADD) assay detects DNA base lesions and strand breaks using an enzymatic cocktail specific for these lesions that removes the lesion and tags the resulting gap or strand break with a modified base for fluorescent detection [27]. The assay was performed as described previously with minor modifications. After permeabilization, cells were incubated with uracil DNA glycosylase (UDG) to remove uracil (NEB #M0304S), formamidopyrimidine [Fapy]-DNA glycosylase (Fapy-DNA glycosylase NEB #M0240S) to remove Fapy lesions, T4 Pyrimidine dimer glycosylase (T4PDG NEB #M-308S) to remove pyrimidine dimer lesions, endonuclease IV (Endo IV NEB #M0304S) to process oxidative damage, AP sites and modifies 3’ phosphates to 3’ OH, and endonuclease VIII (Endo VIII NEB #M0299S) to remove damaged pyrimidines diluted in 1X Thermpol buffer and incubated at 37°C for 1 h. Damage sites are labeled by DNA polymerase I Klenow large fragment (lacking 5’ to 3’ exonuclease activity) incubated with Digoxigenin-11-dUTP, alkali-labile (Dig) (Sigma-Aldrich #DIUTP-RO) at 37°C for 1 h. The Dig-dUTP is covalently incorporated into the DNA for detection of damage sites. Cells were then washed in PBS, blocked using 2% BSA in PBS and Dig was then detected using an anti-Dig antibody (abcam #ab420 clone 21H8) at a dilution of 1:250 in 2% BSA in PBS for 1 h at RT. Samples were then stained with goat anti-Mouse Alexa Fluor 546 at 1:400 in 2% BSA in PBS for 45 min at RT, and nuclei were stained as for immunofluorescence. Image analysis was performed as described previously [27]. MDA-231 cells were used to set the 546 nm laser gain, and the other cells were imaged at that gain. The control experiment was performed with the complete RADD cocktail and Klenow but without the insertion of Dig-dUTP. Antibody staining in this sample was used to ensure the specificity of RADD measurements. A minimum of 200 cells per experiment were analyzed over three biological replicates. The mean nuclear fluorescence intensity was normalized to the MDA-231 cells and reported as ratio of change over MDA-231.

### 355 nm laser microirradiation

Cells were plated as for immunofluorescence in 8-well chambered coverglass at 4 x 10^4^ cells per chamber as described above. The next day, cells were treated with 10 μM bromodeoxyuridine BrdU (Sigma Aldrich #B5002) for 24 hours to sensitize the cells to microirradiation. Microirradiation was performed on a Nikon A1r scanning confocal microscope that was modified to include a 355 nm laser. Experiments were performed using an ultraviolet passing S Fluor 20x/0.75 objective as described previously [28, 29]. Samples were fixed with 3.7% formaldehyde at various time points and processed by immunofluorescence for XRCC1 fluorescence intensity. Intensity levels of damage foci were normalized by subtracting an adjacent region within the nucleus and reported as fluorescence intensity in arbitrary units (a.u.) as described [28].

### Fluorescence multiplex host cell reactivation

Fluorescence multiplex host cell reactivation (FM-HCR) assays were performed as described previously [30, 31]. Briefly, the cells were seeded 48 h before transfection into T25 tissue culture flasks (Thermofisher,156367) and collected for transfection at 85% confluence. Cells were electroporated using the Neon transfection system (ThermoFisher, MPK5000) at 1200 V, and 20 ms, with 2 pulses. Transfected cells were seeded into 12-well culture plates and harvested 24 h after transfection for flow cytometric analysis. Transfection efficiency for each assay is controlled for by the inclusion of an undamaged fluorescent reporter plasmid with the repair reporter constructs as described in [31]. Fluorescent reporter expression is given as the percentage of the fluorescent reporter protein expressed compared to the undamaged control plasmids from a second transfection as described in detail in [30, 31]

### Cytotoxicity

Cytotoxicity was determined with cell growth inhibition assays. Cells were seeded at 3 x 10^4^ cells per well in 6-well dishes. HCC1806 cells were treated the next day, and MDA-157, MDA-231, and MDA-468 were treated after 48 h. All treatments were performed in triplicate with a minimum of 3 replicates each. Cells were treated with carboplatin (Sigma-Aldrich #C2538) or doxorubicin (Selleck Chem #S1208) diluted to 10 mM in DMSO, then diluted in growth medium. The cells were exposed to DMSO vehicle, carboplatin or doxorubicin continuously for 5 days. Methyl methanesulfonate (MMS) (Sigma-Aldrich #129925) or potassium bromate (KBrO_3_) (Sigma-Aldrich #60085) were diluted in growth medium for 1 h exposures. Cells were washed in phosphate buffered saline (PBS, Cellgro #21-031-CV), growth medium was replaced, and the cells were incubated for 5 days. Cells were treated with olaparib (Selleck Chemicals #S1060) diluted to 10 mM in DMSO then diluted in growth medium for 24 hours. Cells were washed with PBS, growth medium was replaced, and the cells were incubated for 5 days. All cells were counted with a Bio-Rad TC20 automated cell counter. Results were normalized to either DMSO vehicle control or medium control exposed cells and graphed to generate values of half maximal inhibitory concentration (IC_50_) values using GraphPad Prism software.

### Statistical analysis

Quantification of fluorescent images was performed in the Nikon Elements software package with a minimum of at least 150–200 cells per experiment, with each experiment performed at least three times. The mean intensity or N/C ratio is reported. IC_50_ values were generated utilizing the GraphPad Prism software with three replicates for each concentration, repeated a minimum of three times, and average IC_50_ values reported. The fluorescent signal generated from the repair of the damaged and undamaged substrates is quantitated in each triplicate, and the mean reported. One-way ANOVA and means were compared with Dunnett post hoc analysis. Comparison groups are indicated after values. All means are reported ± standard error of the mean (SEM).

## Results

### XRCC1 expression in the TCGA

**Fig 1:**
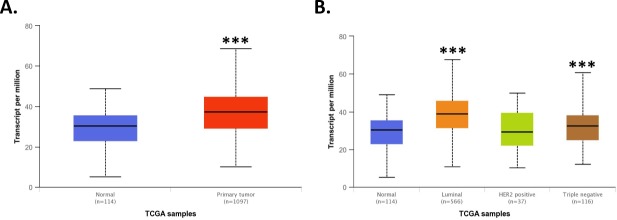
TCGA analysis of XRCC1. A) TCGA analysis using the UALCAN web interface [23] revealed XRCC1 transcript expression to be significantly higher in breast tumor tissue over normal tissue (p = 1.6 x 10^−12^, Primary tumor to Normal). B) XRCC1 was increased in Luminal (p < 1 x 10^−12^, Luminal to Normal) and TNBC (p < 0.001, TNBC to Normal) tumor types compared to normal tissue using the same analysis interface. *** P < 0.001.

The TCGA Breast Invasive Carcinoma dataset was analyzed using the UALCAN data portal [23] for XRCC1 expression. Despite previous publications indicating a loss in XRCC1 to be an important indicator for TNBC [16], XRCC1 transcript expression levels were significantly higher in primary tumor samples than in normal tissues with a p value of 1.6 x 10^−12^ (Primary tumor to Normal)(Fig 1A). Further analysis of XRCC1 expression across breast cancer types shows increased expression of XRCC1 in Luminal (p < 1 x 10^−12^, Luminal to Normal) and TNBC (p < 0.001, TNBC to Normal) tumor types (Fig 1B), but not in HER2 positive tumors.

### Base excision repair protein expression

**Fig 2:**
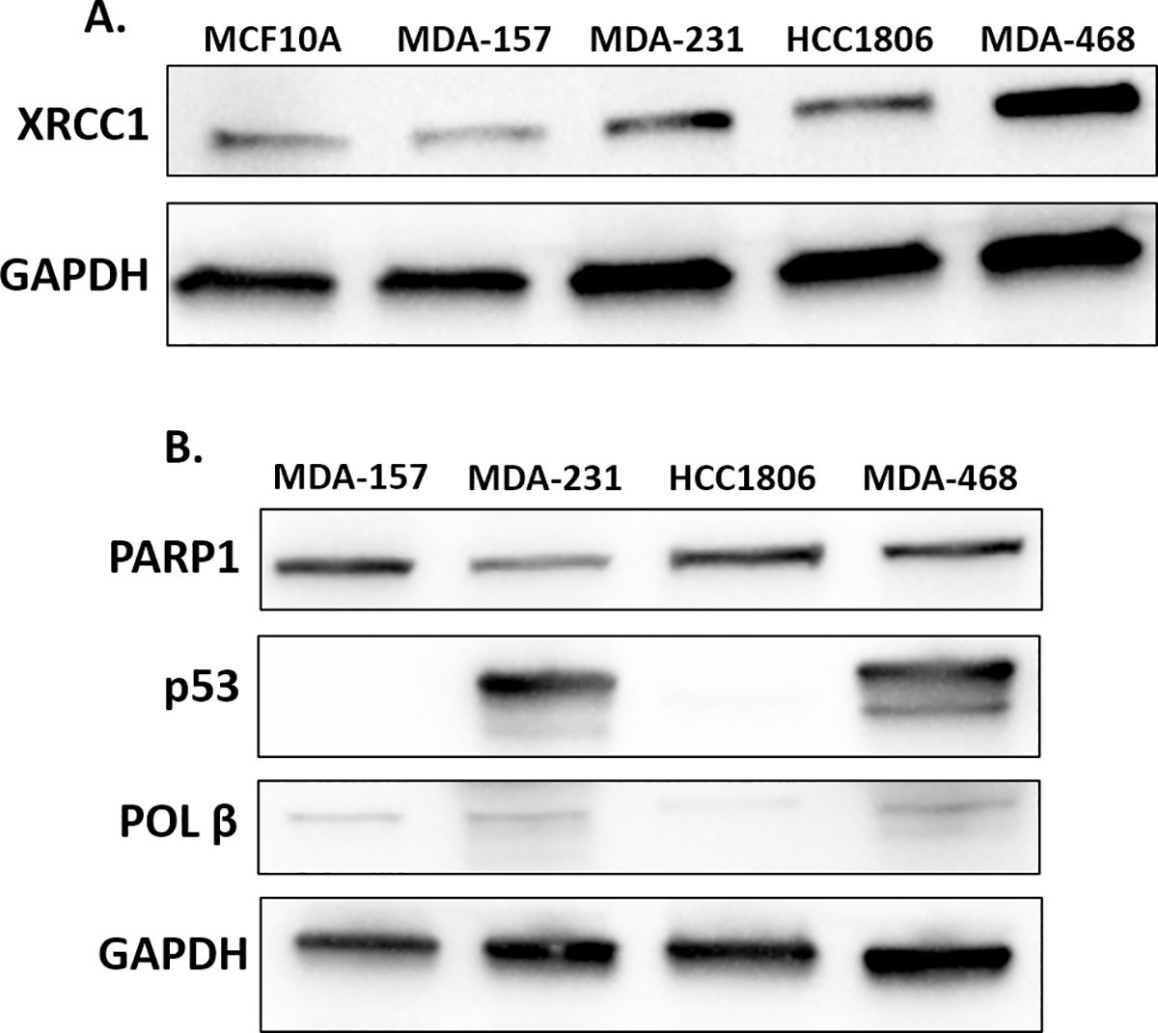
Base excision repair proteins in TNBC cell lines. A) Lysates of MCF10A, MDA-157, MDA-231, HCC1806, and MDA-468 were probed by immunoblot for the expression of XRCC1 with GAPDH serving as loading control. B) Lysates of MDA-MB-157 (MDA-157), MDA-MB-231 (MDA-231), HCC1806, and MDA-MB-468 (MDA-468) were probed by immunoblot for the expression of PARP1, p53, POL β, and GAPDH as a loading control.

To further examine XRCC1 expression and the expression of other key DNA repair proteins *in vitro*, we utilized four highly characterized TNBC cell lines: MDA-157, MDA-231, HCC1806, and MDA-468 [32–34]. Levels of XRCC1 expression were lowest in MDA-157 cells similar to non-tumorigenic epithelial MCF10A cells, higher in MDA-231 and HCC1806 cells, and the highest in MDA-468 cells (Fig 2A, quantified in S1A Fig). The expression levels of PARP1 were moderate and similar in MDA-157, HCC1806, and MDA-468 but lower in MDA-231 cells (Fig 2B). We observed low POL β protein levels in all cell lines. MDA-231 and MDA-468 had high levels of p53 due to gain-of-function stabilizing mutations that also promote tumorigenesis [35, 36]. No p53 protein was detected in MDA-157 cells, and low levels were detected in HCC1806 cells; both cell lines contain truncating mutations in p53 (Fig 2B, S1A Fig) [37–40]. We observed an increased expression pattern in BRCA1 across the cell lines with MDA-468 having the highest expression of BRCA1 (S1B Fig). There are no reported BRCA1 mutations in these cell lines [33]. Variation in the expression of BER proteins are observed across the four TNBC model cell lines, with XRCC1 showing increased expression over the non-tumorigenic epithelial MCF10A cells, consistent with the TCGA trends.

### Subcellular localization of critical base excision repair proteins

For effective DNA repair and maintenance of genomic fidelity, the proteins involved must be localized in the nucleus. We examined the expression and nuclear localization of key BER interactors XRCC1 and POL β using immunofluorescence. XRCC1 has primarily nuclear localization, which is consistent with its DNA repair functions [25]. POL β functions in both nuclear and mitochondrial BER and is observed within the cytoplasm, mitochondria, and nucleus of cells [26, 41].

**Fig 3:**
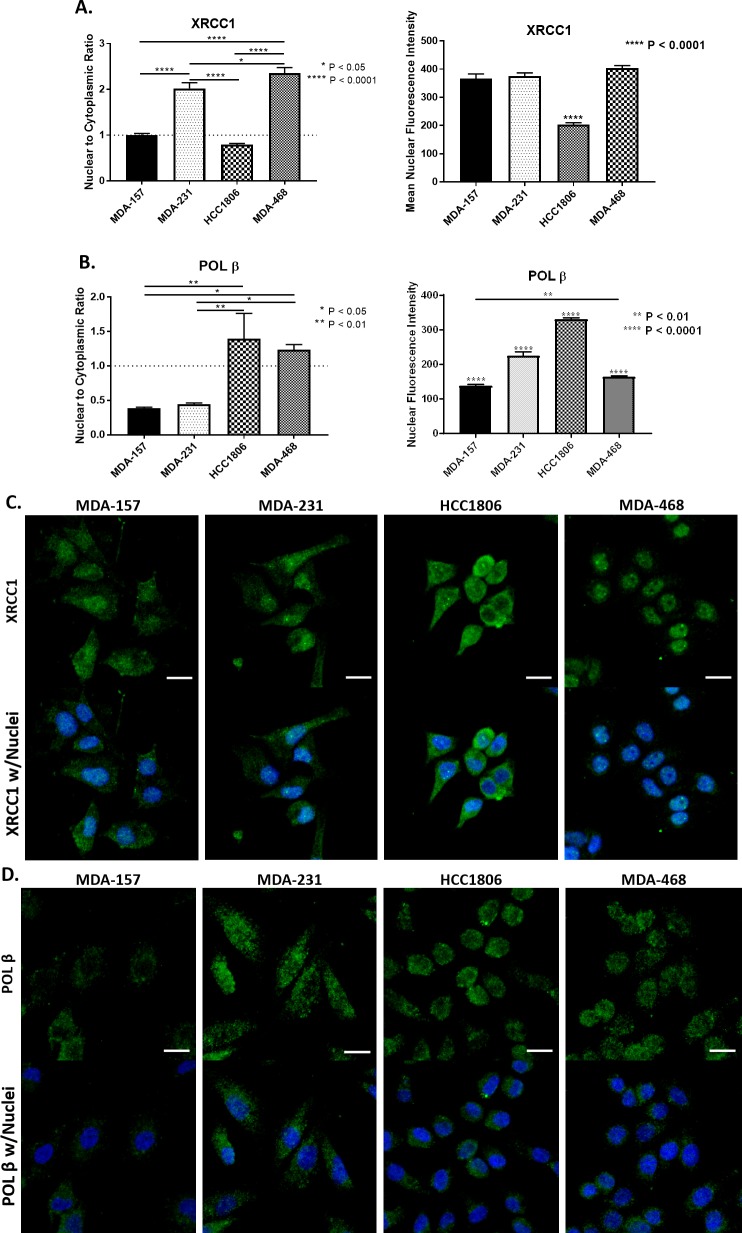
Subcellular localization and quantification of BER proteins. A) Left, XRCC1 nuclear to cytoplasmic ratio (N/C) is reported as 1 representing equal distribution (MDA-157), > 1 representing nuclear localization (MDA-231 and MDA-468), and < 1 representing nuclear exclusion (HCC1806). (**** P < 0.0001, MDA-157 to MDA-231, MDA-157 to MDA-468, MDA-231 to HCC1806, HCC1806 to MDA-468; * P < 0.05, MDA-231 to MDA-468). Right, mean fluorescence intensity of XRCC1. (**** P < 0.0001, HCC1806 to MDA-157, MDA-231, and MDA-468) B) Left, N/C ratio of POL β. (** P < 0.01, MDA-157 to HCC1806, MDA-231 to HCC1806; * P < 0.05, MDA-157 to MDA-468, MDA-231 to MDA-468) Right, mean fluorescence intensity of POL β. (**** P < 0.0001, MDA-157 to MDA-231, MDA-157 to HCC1806, MDA-231 to HCC1806, MDA-231 to MDA-468, HCC1806 to MDA-468; ** P < 0.01, MDA-157 to MDA-468) C) Representative images of localization of XRCC1 in TNBC cell lines. D) Representative images of localization of POL β in TNBC cell lines. Scale bar = 20 μm.

We assessed the nuclear to cytoplasmic ratio (N/C) of XRCC1 and POL β, with a value of 1 representing equal distribution between the nucleus and cytoplasm, while a value of less than one represents exclusion from the nucleus, and a value greater than one represents predominantly nuclear localization. The MDA-157 cells exhibited a relatively equal distribution of XRCC1 between the nucleus and cytoplasm with an N/C ratio of 1.01 ± 0.03 (Fig 3A and 3C and S2 Fig). MDA-231 showed higher nuclear content of XRCC1 with an N/C ratio of 2.02 ± 0.12, though some cytoplasmic content is still observed (Fig 3A and 3C, S2 Fig). HCC1806 cells had partial exclusion of XRCC1 from the nucleus with an N/C ratio of 0.79 ± 0.02 (Fig 3A and 3C, S2 Fig). MDA-468 cells had the highest N/C ratio of the cell lines tested (2.36 ± 0.12) with XRCC1 almost exclusively in the nucleus (Fig 3A and 3C, S2 Fig). All N/C ratios of XRCC1 were significantly different when compared with each other with the exception of MDA-157 compared with HCC1806. Examination of the mean nuclear intensity of XRCC1 within these cell lines shows similar amounts of XRCC1 are localized into the nucleus for MDA-157, MDA-231, and MDA-468 (Fig 3A right), so the changes in N/C ratio are driven by increasing amounts of XRCC1 within the cytoplasm for MDA-157 and MDA-231 (Fig 3A and 3C). While the decreased mean nuclear intensity of XRCC1 in the HCC1806 confirmed the cytoplasmic level of XRCC1 was higher than the nuclear (Fig 3A and 3C). The N/C ratio of XRCC1 was confirmed by immunoblot following subcellular fractionation for each cell line (S2 Fig).

Some studies have suggested POL β localization and protein stability is dependent on XRCC1. Given the diminished nuclear localization of XRCC1 in HCC1806 cells, we also examined the nuclear content of POL β across the cell lines. Consistent with immunoblot results, POL β staining was low across all cell lines despite high levels of XRCC1 (Fig 3B and 3D, S2 Fig). Localization of POL β to the nucleus was also variable between cells (Fig 3B and 3D, S2 Fig). Low nuclear content of POL β was observed in MDA-157 and MDA-231 cells with N/C ratios of 0.39 ± 0.02 and 0.45 ± 0.02, respectively (Fig 3B). HCC1806 showed significantly higher nuclear enrichment of POL β with an N/C of 1.40 ± 0.37, despite XRCC1 being predominately cytoplasmic when compared with both MDA-157 and MDA-231 (Fig 3B and 3D). MDA-468 cells also showed significantly enriched nuclear POL β with an N/C of 1.24 ± 0.08 when compared to both MDA-157 and MDA-231. Examination of the mean nuclear fluorescent intensity showed higher mean fluorescence intensity of POL β in the nucleus of HCC1806, suggesting the majority of the protein is localized there (Fig 3B). Even though MDA-157, MDA-231, and MDA-468 show different mean fluorescent intensities of POL β, the differences are small compared to the HCC1806, so the changes in N/C ratios in these cell lines is driven by the cytoplasmic content of the POL β (Fig 3B).

**Fig 4:**
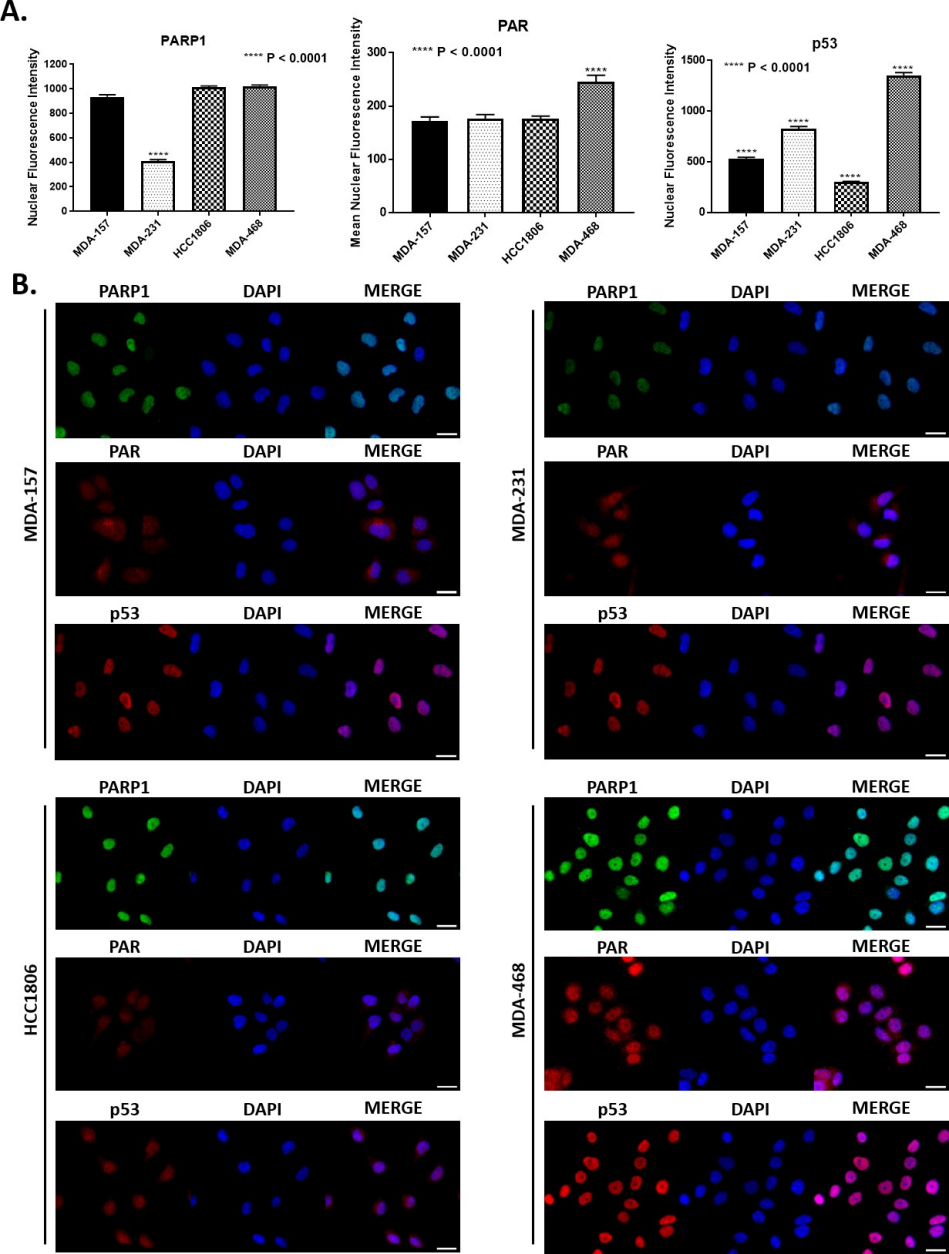
Nuclear intensity of key BER proteins. A) Quantification of mean nuclear fluorescence intensity is shown for PARP1 (**** P < 0.0001, MDA-231 to MDA-157, HCC1806, and MDA-468), PAR (**** P < 0.0001, MDA-468 to MDA-157, MDA-231, and HCC1806), and p53 (**** P < 0.0001, MDA-157 to MDA-231, MDA-157 to MDA-468, MDA-157 to HCC1806, MDA-231 to MDA-468, MDA-231 to HCC1806, and MDA-468 to HCC1806). B) Representative fluorescent images used for quantification in A for PARP1, PAR, and p53 for all cell lines tested. Scale bar = 20 μm.

Given the unexpected cellular distribution of key BER factors XRCC1 and POL β, we also examined cellular levels of PAR, which is polymerized by PARP1 at sites of DNA damage and increases in the presence of BER defects [42, 43]. Nuclear levels of PAR were largely consistent, with only the MDA-468 cells having significantly higher nuclear intensity of PAR. The HCC1806 cells has similar PAR levels to MDA-157 and MDA-231, despite XRCC1 being largely cytoplasmic in these cells (Fig 4A).

We then examined the nuclear content of PARP1 and p53. MDA-157 contained roughly 2.4-fold more nuclear PARP1 than MDA-231 cells. HCC1806 had 1.6-fold and MDA-468 1.9-fold more nuclear PARP1 than MDA-231 cells (Fig 4A). PARP1 highly localized to the nucleus in all cell lines, yet expression levels were highly variable across all cell lines, consistent with immunoblotting (Figs 2B and 4B). We observed that p53 nuclear localization was consistent with mutational status, with HCC1806 and MDA-157 displaying significantly low nuclear staining, in contrast with strong nuclear staining of p53 in MDA-231 and MDA-468 cells, consistent with immunoblot data in Fig 2. Representative images of all cell lines are shown in Fig 4B, with PARP1 shown in green, while PAR and p53 are shown in red.

Significant differences in the nuclear localization of PARP1, XRCC1 and POL β are observed across the TNBC cell lines. MDA-468 has the most “normal” distribution of PARP1, XRCC1 and POL β of the cell lines with nuclear enrichment of both factors. MDA-157 and MDA-231 showed high cytoplasmic content for both XRCC1 and POL β and high levels of PAR, suggesting deficiencies in BER may exist in these cell lines. HCC1806 is the most defective in XRCC1 nuclear localization, though PARP1 and POL β are highly enriched.

### Basal levels of DNA damage in TNBC cell lines

**Fig 5:**
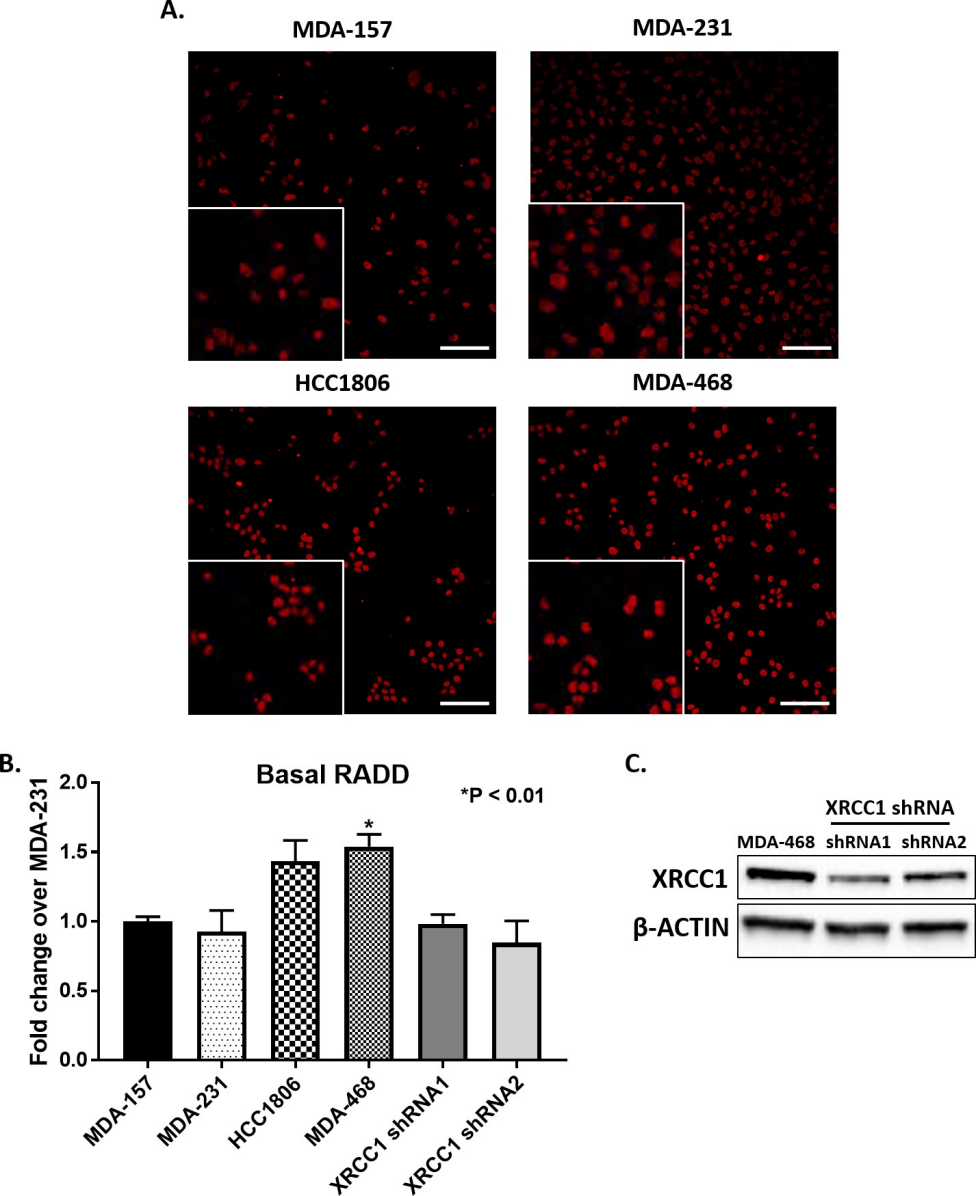
Basal DNA damage in TNBC cell lines. A) Representative images of basal levels of DNA damage as measured by RADD in MDA-157, MDA-231, HCC1806, MDA-468 with insets showing blown up cropped images. Scale bar = 100 μm. B) Quantification of images in A normalized to MDA-231 show an increase in DNA damage in HCC1806, and a significantly higher amount (P < 0.01, MDA-468 to MDA-231) of DNA damage for MDA-468. MDA-468 cells with XRCC1 knockdown show decreased levels of basal DNA damage. C) Expression of XRCC1 in MDA-468 and MDA-468 XRCC1 knockdown cells.

Based on the altered protein expression and localization of BER proteins in TNBC cells, we evaluated the cells for variation in levels of basal DNA damage using the broad spectrum DNA damage assay, Repair Assisted Damage Detection (RADD) [27]. RADD uses bacterial DNA glycosylases to detect and excise abasic sites, oxidative base lesions, bulky DNA adducts, and thymine dimers (see Material and Methods). Then the gaps in the DNA where the lesions were excised or strand breaks occur are tagged by Klenow polymerase with a Digoxigenin-11-dUTP. Damage sites are detected by immunofluorescence as described in the materials and methods [27]. RADD was applied to the TNBC cell panel, and the nuclear fluorescence intensity of basal DNA damage quantified (Fig 5, S3 Fig). MDA-157 and MDA-231 cells had similar low levels of basal DNA damage (Fig 5B). However, HCC1806 cells had 1.4-fold more DNA damage over MDA-231 (P = 0.09, HCC1806 to MDA-231) and MDA-468 cells had 1.5-fold more damage than MDA-231 cells (P < 0.01, MDA-468 to MDA-231) (Fig 5A). Representative images of nuclear fluorescence for all cell lines are shown in Fig 5A. Knockdown of XRCC1 utilizing two distinct shRNA constructs in MDA-468 cells (Fig 5C) shows a decrease in the basal amounts of DNA damage (Fig 5B), indicating that excess XRCC1 plays a role in higher levels of basal DNA damage.

### XRCC1 recruitment to single-strand breaks is altered in TNBC cells

The varied levels of basal DNA damage seen in Fig 5 and altered expression and localization of XRCC1 in Fig 2 and Fig 3 indicated that DNA repair functions of XRCC1 may be altered in TNBC cells. As XRCC1 has no known enzymatic activity, we assessed DNA repair activity by examining the recruitment and retention of XRCC1 at sites of DNA damage induced by laser microirradiation. Single-strand breaks were induced in a subnuclear region by microirradiation with a 355 nm laser, and we assessed XRCC1 recruitment and retention to the damage site at various time points after inducing damage [28, 29].

**Fig 6:**
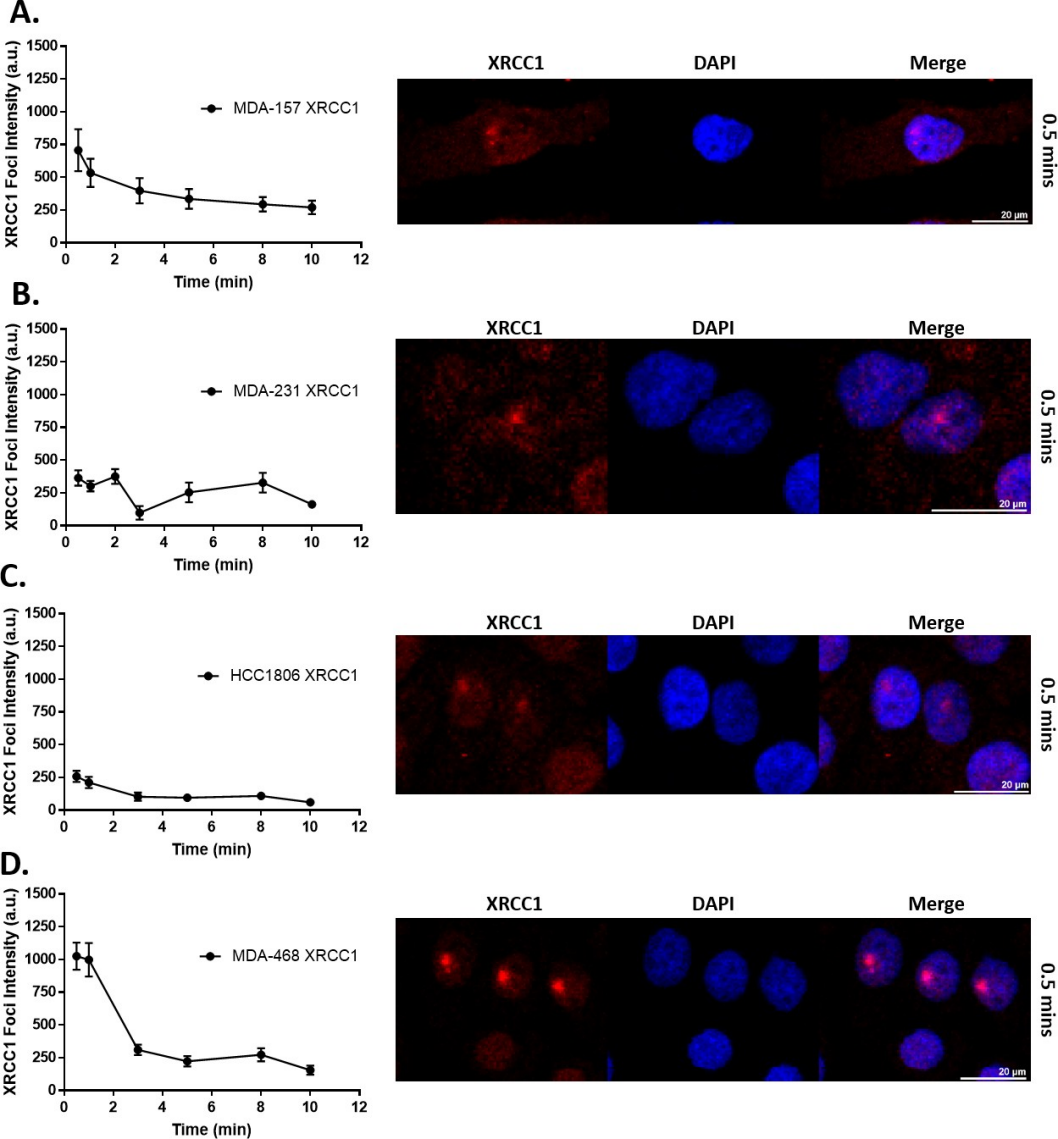
XRCC1 recruitment following 355 nm laser microirradiation in TNBC cell lines. Microirradiation with a 355 nm laser occurred and cells were fixed at the indicated time points and processed for immunofluorescence of XRCC1. Quantification over time is shown on the left with a representative image from 0.5 min shown on the right for A) MDA-157, B) MDA-231, C) HCC1806, and D) MDA-468. A minimum of 18 cells were irradiated over three separate experiments and quantified as described in the materials and methods section and reported as XRCC1 Foci Intensity in arbitrary units (a.u.). Scale bar = 20 μm.

As shown in Fig 6, XRCC1 recruitment over time is graphed on the left, with corresponding images of peak XRCC1 recruitment foci shown on the right. XRCC1 recruited to the DNA damage site in MDA-157 cells with an average intensity of 707 a.u. and dissociated within 5 min, with 50% retention at 1.7 min (Fig 6A). In contrast, MDA-231 cells had two XRCC1 recruitment peaks at 364 a.u. within 0.5 min that resolved by 3 min and at 8 min, respectively (Fig 6B). XRCC1 weakly recruited to the damage site in HCC1806, peaking only at 258 a.u. (Fig 6C). In MDA-468 cells, XRCC1 recruitment was rapid and robust, peaking at 1025 a.u. within 0.5 min (Fig 6D). XRCC1 was rapidly dissociated from the damage site with 50% retention at 2.2 min and complete resolution of the damage foci within 3 min of microirradiation (Fig 6D). Double-strand breaks (DSBs) were absent in all cell lines as confirmed by the lack of co-localization of DSB markers 53BP1 and γH2AX at 10 min after microirradiation, except for a slight increase of γH2AX at 10 min in HCC1806 cells which displayed weak XRCC1 recruitment, likely due to XRCC1’s cytoplasmic localization (Fig 3, S5 Fig, S6 Fig).

### Base excision repair capacity is altered in TNBC cells

**Fig 7:**
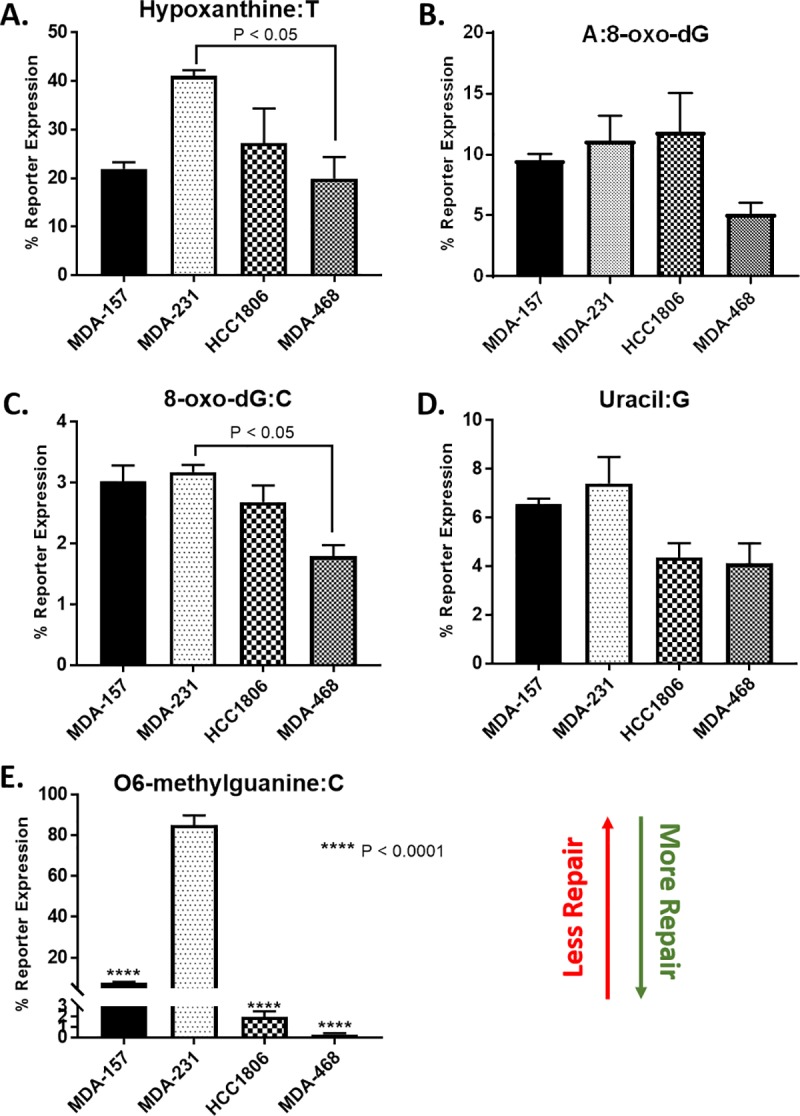
FM-HCR analysis of DNA repair capacity in TNBC cell lines. Cells were transfected with fluorescent reporter plasmids containing the indicated DNA lesions, A) Hypoxanthine:T (P < 0.05, MDA-231 to MDA-468), B) A:8-oxo-dG, C) 8-oxo-dG:C (P < 0.05, MDA-231 to MDA-468), D) Uracil:G, E) O6-methylguanine:C (**** P < 0.0001, MDA-157 to MDA-231, HCC1806 to MDA-231, MDA-468 to HCC1806), as well as an undamaged plasmid to normalize for transfection efficiency. DNA repair capacity is inversely proportional to % reporter expression.

Given the BER defects observed through expression, localization, and response to induced DNA damage, we utilized a flow cytometric host cell reactivation assay (FM-HCR) to assess the repair capacities of TNBC cells across BER substrates [30, 31]. The FM-HCR assays measured DNA repair capacity using transiently transfected fluorescent reporter plasmids. By reporting repair of each DNA lesion in a different fluorescent channel, simultaneous measurements were made for multiple DNA repair pathways to assess the diverse BER capacity in TNBC cells. The BER reporters include GFP_hypoxanthine:T, which primarily reports AAG (also known as MPG) glycosylase-mediated excision of hypoxanthine opposite thymine (Fig 7A); mPlum_A:8-oxo-dG, which reports MUTYH glycosylase catalyzed excision of adenine opposite 8-oxo-2'-deoxyguanosine (8-oxo-dG) (Fig 7B); mOrange_8-oxo-dG:C, which reports the activity of several glycosylases (OGG1, NEIL1, NEIL2) that excise 8-oxo-dG opposite cytosine (Fig 7C); and BFP_Uracil:G, which primarily reports UNG glycosylase catalyzed excision of uracil opposite guanine (Fig 7D). mPlum_O^6^-methylguanine:C was used to assess the removal of O^6^-methylguanine opposite thymine by MGMT (Fig 7E).

In the selected TNBC cells, MDA-468 cells were the most BER proficient with efficient repair observed for all BER substrates. MDA-468 also efficiently repaired O^6^-methylguanine by direct reversal. MDA-231 cells were defective for repair of O^6^-methylguanine, and overall the least proficient at repairing BER substrates. MDA-157 cells repaired most BER substrates efficiently except for 8-oxo-dG:C and uracil. Intermediate repair efficiency for O^6^-methylguanine was observed for MDA-157. HCC1806 cells repaired O^6^-methylguanine efficiently but had low repair capacity for all BER substrates except uracil, for which the repair proficiency matched MDA-468 cells.

### DNA damaging agents induce selective cytotoxicity in TNBC cells

We then analyzed the sensitivity of the four TNBC cell lines to clinical DNA damaging agents. Cytotoxicity of carboplatin and doxorubicin have been widely reported for these cell lines with a large range of IC_50_ values observed utilizing various methods. BER proteins, including XRCC1, are involved in the repair of the platinum-DNA adducts, strand breaks, and DNA-protein crosslinks induced by these agents, though other repair pathways are more dominant [44–47]. TNBC cell line sensitivity was determined by growth inhibition following continuous exposure to carboplatin and doxorubicin (Table 1). MDA-468 were the most sensitive to both carboplatin and doxorubicin compared with the other cell lines tested, consistent with previously reported data on these cell lines with platinum-based therapies and doxorubicin [34, 48, 49].

**Table 1:** Sensitivity of TNBC cell lines to DNA damaging agents.

Cell Line	Carboplatin IC_50_ (μM)	Doxorubicin IC_50_ (nM)	MMS IC_50_ (mM)	Olaparib IC_50_ (μM)	KBrO_3_ IC_50_ (mM)
MDA-157	9.53 ± 1.12	10.11 ± 1.05	0.75 ± 0.07	6.2 ± 2.1	22.56 ± 3.55
MDA-231	7.62 ± 0.79	18.50 ± 6.83	0.28 ± 0.02	> 25	24.98 ± 1.91
HCC1806	1.11 ± 0.07	17.90 ± 1.00	0.61 ± 0.07	2.22 ± 0.46	16.22 ± 2.13
MDA-468	1.01 ± 0.07	10.52 ± 0.67	1.84 ± 0.10	5.88 ± 2.27	11.87 ± 1.01

We also determined TNBC cell line sensitivity to the PARPi olaparib. PARP1 recognizes abasic sites and single-strand breaks and synthesizes poly(ADP-ribose) (PAR) polymers to recruit additional BER proteins such as XRCC1, POL β, and DNA LIG3 for repair [8, 21]. Olaparib inhibits PAR production thereby reducing BER mediating repair [46, 50]. Cells were treated with increasing concentrations of olaparib up to 25 μM for 24 h, and cytotoxicity determined by growth inhibition. MDA-231 cells were most resistant to olaparib treatment up to the highest concentration tested of 25 μM, despite BER defects (Fig 7, Table 1). MDA-157 and MDA-468 cells were sensitive to olaparib treatment with IC_50_ values of 6.2 and 5.88 μM, respectively, while HCC1806 cells were the most sensitive to olaparib treatment with an IC_50_ of 2.22 μM (Table 1).

BER is also responsible for the repair of oxidative DNA lesions [51, 52]. TNBC cell sensitivity to the oxidizing agent potassium bromate (KBrO_3_ [51]) was tested at increasing concentrations up to 25 mM. MDA-231 and MDA-157 exhibited IC_50_ values of 24.98 mM and 22.56 mM, respectively, with no significant difference between the two. HCC1806 cells were sensitive to KBrO_3_ with an IC_50_ value of 16.22 mM representing a significant difference compared to MDA-231 (P < 0.05, HCC1806 to MDA-231) (Table 1). MDA-468 cells were also sensitive to KBrO_3_ with an IC_50_ of 11.87 mM representing a significant difference compared to MDA-231 cells (P < 0.01, MDA-468 to MDA-231) (Table 1).

Finally, we determined TNBC cell line sensitivity to the DNA methylating agent methyl methanesulfonate (MMS). BER is responsible for the resolution of alkylation base damage induced by MMS, and XRCC1-deficient cells are hypersensitive to MMS [53]. We determined the MMS sensitivity (IC_50_) of the four TNBC cell lines. MDA-468 cells were the most resistant to MMS with an IC_50_ of 1.84 mM, followed by MDA-157 (0.75 mM) and HCC1806 cells (0.61 mM). MDA-231 cells were the most sensitive to MMS with an IC_50_ of 0.28 mM (Table 1). Knockdown of XRCC1 by two distinct shRNAs in the MDA-468 cell line sensitized these cells to growth inhibition by MMS (S8 Fig), confirming the role that XRCC1 plays in the repair of alkylation by BER.

## Discussion

DNA repair defects are increasingly focused on for clinical assessments with gene expression panels and protein biomarkers sought to predict patient response and improve therapeutic outcomes. However, characterization of DNA repair pathways is often lacking in preclinical models and is essential for understanding the effects of DNA repair defects on the efficacy of therapeutic agents. Several studies have noted deficiency in XRCC1 in breast cancers and proposed defects in BER as targets for therapeutic intervention [16, 18, 19]. However, little is known about BER defects in breast cancer, particularly those linked to XRCC1. Given the aggressiveness of TNBC and the poor treatment options for recurrent TNBC, identifying and characterizing BER defects, which may promote resistance to standard of care treatment is critical.

While expression changes in PARP1 and POL β occur in TNBC, these expression changes have failed to serve as effective biomarkers for therapeutic response [1–3]. So we evaluated XRCC1 expression in the TCGA Breast Invasive Carcinoma dataset using the UALCAN data portal [23]. Unlike previous reports, over-expression of XRCC1 was observed in this dataset similar to the over-expression observed in the four preclinical models used in this study. XRCC1 transcript expression levels were higher in tumor samples than in normal tissues, and in Luminal and TNBC subtypes compared with normal tissue. Pathology images from the TCGA dataset also showed high protein content in breast carcinomas consistent with the high transcript levels [54]. Therefore, XRCC1 over-expression may be more relevant to examining DNA repair defects in TNBC than XRCC1 deficiency.

To explore this hypothesis, we examined XRCC1 in four TNBC preclinical models to determine if XRCC1 expression changes account for varied responses to DNA damaging agents frequently observed [32–34]. XRCC1 protein levels varied across the four common preclinical model cell lines, and more importantly, XRCC1 localization also varied among the cell lines. XRCC1’s function as a scaffold protein in BER is well described, as is its localization into the nuclear compartment [25, 55, 56]. There is no evidence that XRCC1 is involved in mitochondrial BER, and mislocalization of XRCC1 into the cytoplasm by the single nucleotide polymorphism R280H has been associated with defective nuclear BER [57–59]. While the TNBC cell lines used in this study do not contain the R280H polymorphism, we observed significant cytoplasmic content of XRCC1 that correlated with BER defects measured by micro-irradiation and FM-HCR [60]. HCC1806 had low nuclear and high cytoplasmic content, which resulted in low recruitment and retention of XRCC1 to laser-induced DNA damage and low repair of all BER substrates except uracil in the FM-HCR assay. MDA-157 and MDA-231 also showed cytoplasmic localization of XRCC1, low repair of some BER substrates and altered XRCC1 recruitment profiles, despite having sufficient levels of nuclear XRCC1 (Fig 3A right). Only MDA-468 with the almost exclusive nuclear localization of XRCC1 was highly proficient at BER (Fig 7).

In addition to being a critical component in BER, XRCC1 has been proposed to stabilize the expression of POL β and participate in POL β nuclear localization [43, 61, 62], With the altered expression and localization of XRCC1, we examined POL β expression and localization in the TNBC cell models. POL β expression also varied between the cell lines, with higher XRCC1 levels correlating with higher POL β protein content. However, the TNBC cells also showed altered localization of POL β that did not correlate with altered XRCC1 localization. The MDA-157 and MDA-231 cells showed low nuclear content of POL β, whereas HCC1806 and MDA-468 showed POL β nuclear enrichment [26, 41]. Loss of POL β does not induce the same level of hypersensitivity to DNA damaging agents as loss of XRCC1 [53, 63, 64]. However, loss of nuclear POL β may reduce the efficiency of repair and likely contributes to reduced BER capacity and sensitivity of MDA-157and MDA-231 cells to DNA damaging agents (Fig 7, Table 1).

Defective repair leaves DNA damage within the genome promoting mutation and instability. To observe the consequences of DNA repair defects, we measured DNA damage levels in the TNBC cells using a broad spectrum DNA lesion detection assay [27]. Expression levels of XRCC1 (Fig 2) correlated with increased levels of basal DNA damage. Low levels of XRCC1 in MDA-157 and MDA-231 cells corresponded to low levels of basal DNA damage, similar to that in the non-tumorigenic epithelial cell line MCF10A (S3 Fig). However, HCC1806 and MDA-468 cells had increased XRCC1 levels that corresponded to higher levels of DNA damage (Fig 5). We expected high levels of DNA damage in HCC1806 cells, where XRCC1 is mislocalized to the cytoplasm. However, the high levels of DNA damage in MDA-468 cells, and the lack of known *BRCA1* mutations and dependence on mutant p53 for survival [36] were surprising. Other DNA repair defects in the MDA-468 cells may contribute to the DNA damage level. However, knockdown of XRCC1 in the MDA-468 significantly decreased basal levels of DNA damage, suggesting that elevated levels of on-going repair from increased expression of XRCC1, PARP1, and POL β contribute to the high basal levels of DNA damage.

Elevated levels of BER in MDA-468 cells are consistent with the proficiency of these cells to repair BER substrates (Fig 7) by the rapid and robust recruitment of XRCC1 to laser-induced DNA damage (Fig 6), and the resistance of these cells to MMS (Table 1). The recruitment of XRCC1 was greater in MDA-468 than MDA-157 and HCC1806 cells, in which defects in XRCC1 expression and localization were observed. MDA-468 cells also recruited XRCC1 more robustly than MDA-231 cells, which had two recruitment waves of XRCC1 similar to a recent report examining XRCC1 in U2OS cells [65]. Interestingly, the second wave of XRCC1 recruitment observed in the U2OS cells was observed after CRISPR editing made the cell XRCC1 deficient and ectopic expression of XRCC1 was introduced. It is unclear if the second wave of recruitment in MDA-231 is due to inefficient recruitment of POL β to the damage site from the low nuclear content of these cells (Fig 6) [43], or could possibly be due to a more complex BER pathway that is needed for correct end-tailoring of nicked DNA before gap-filling by the polymerase [28]. Further work is needed to better understand these recruitment dynamics.

The inefficiency in BER repair by MDA-231 cells was confirmed by the FM-HCR assays (Fig 7) and high sensitivity of MDA-231 cells to MMS (Table 1). The MDA-231 cells were not sensitive to olaparib alone and the least sensitive to KBrO_3_ of the lines tested, despite defects in BER (Table 1). The lack of sensitivity to olaparib may result from the lower levels of its target PARP1 in the MDA-231 cells (Fig 2), while the lack of sensitivity to KBrO_3_ is likely due to the fact that while the MDA-231 cells are the least efficient in BER, XRCC1 is still able to recruit to sites of damage and conduct SSBR (Fig 6). As the levels of DNA damage observed in this cell line are more comparable to the more BER competent MDA-157, low levels of PARP1 and the gain-of-function mutation in p53 observed in MDA-231 cells likely promote alternative repair pathways, like NHEJ, to reduce basal damage levels (Fig 5) [33]. Competition or ineffective signaling by these alternative repair pathways also may explain the altered recruitment profile for XRCC1 in the MDA-231 cells.

Although MDA-468 and MDA-231 cells represented the extremes of BER competencies, the observed sensitivity of MDA-157 and HCC1806 cells to MMS likely resulted from the observed defects in localization of BER proteins, which has not been previously assessed (Fig 3).

Characterization of XRCC1 expression in these preclinical models demonstrates that protein expression, localization, and the basal DNA damage levels are important indicators of DNA repair capacities. Despite the high proficiency for BER observed by FM-HCR, MDA-468 cells are well documented to be sensitive to DNA damaging agents [32, 49, 66]. Over-repair by the BER pathway and high basal DNA damage levels in these cells may generate too many cytotoxic intermediates that promote cell death and increase cellular sensitivity to DNA damaging agents, such as KBrO_3_ (Table 1), rendering these cells an ineffective model for drug evaluation. The present data also demonstrate that defects in BER are prominent in the commonly used TNBC preclinical models. These BER defects have not been characterized or examined previously and need to be investigated in patient tumor samples.

In summary, XRCC1 expression and localization may vary and serve as critical indicators of BER defects in TNBCs and likely other cancers. However, the expression of a single protein may not determine the true extent of DNA repair defects. Our work demonstrates the importance of assessing expression, basal levels of DNA damage, and protein localization in cells and tissues may better predict treatment outcomes when DNA repair defects are present.

## Supporting information

S1 Fig A) Immunoblots from Fig 1A, Fig 1B, and S1B Fig were quantified, normalized to loading control, and reported as fold change relative to MDA-231. ND = no signal detected. B) Lysates for MDA-157, MDA-231, HCC1806, and MDA-468 were probed for BRCA1, with GAPDH serving as a loading control.
(TIF)

S2 Fig Subcellular fractionation of MDA-157, MDA-231, HCC1806, and MDA-468. Cytoplasmic and nuclear fractions of all cells were probed for XRCC1. β-ACTIN was used as a loading control for the cytoplasmic fraction while LAMIN A/C was used as a loading control for the nuclear fraction.
(TIF)

S3 Fig Basal DNA damage in TNBC cell lines in comparison to MCF10A. A) Replotting data from Fig 5B in the presence of MCF10A shows similar levels of DNA damage to that of MDA-157 and MDA-231. (P = 0.09, HCC1806 to MDA-231; * P < 0.01, MDA-468 to MDA-231) B) Representative images of basal levels of DNA damage as measure by RADD including MCF10A. Scale bar = 100 μm.
(TIF)

S4 Fig MDA-157, MDA-231, HCC1806, MDA-468, and MDA-468 XRCC1 shRNA cell lines were tested by immunofluorescence for the presence of γ-H2AX and 53BP1 as indicators of strand breaks. This data indicates that strand breaks are not significantly different in MDA-468 cell lines compared to MDA-468 XRCC1 shRNA cell lines further confirming the ability of RADD to detect broad spectrum DNA damage.
(TIF)

S5 Fig Double strand break markers post microirradiation. DSB markers 53BP1 (Green) and γ-H2AX (Violet) were stained by immunofluorescence at 10 min after micro-irradiation and representative images are shown for A) MDA-157, B) MDA-231, C) HCC1806, and D) MDA-468. Scale bar = 20 μm.
(TIF)

S6 Fig Fluorescence intensity of double-strand break markers from S4 Fig. A) Foci Intensity for γ-H2AX (Left), and 53BP1 (Right) for MDA-157, MDA-231, HCC1806, and MDA-468. B) Mean ± SEM for γ-H2AX and 53BP1 from S5A Fig.
(TIF)

S7 Fig FM-HCR analysis from Fig 6 including the non-tumorigenic cell line MCF10A. A) Hypoxanthine:T (P < 0.05, MDA-231 to MDA-468), B) A:8-oxo-dG, C) 8-oxo-dG:C (P < 0.05, MDA-231 to MDA-468), D) Uracil:G (P < 0.05, MCF10A to HCC1806, MCF10A to MDA-468), E) O6-methylguanine:C (**** P < 0.0001, MCF10A to MDA-231, MDA-157 to MDA-231, HCC1806 to MDA-231, MDA-468 to HCC1806), as well as an undamaged plasmid to normalize for transfection efficiency. DNA repair capacity is inversely proportional to % reporter expression.
(TIF)

S8 Fig A) MMS sensitivity graphs for MDA-468, MDA-468 XRCC1 shRNA1, and MDA-468 XRCC1 shRNA2. XRCC1 shRNA2 showed significantly more cell death at 0.5 mM MMS compared to MDA-468, while at 1.0 mM MMS both XRCC1 shRNA1 and XRCC1 shRNA2 showed significantly more cell death compared to MDA-468. (* P < 0.05, 0.5 mM MMS XRCC1 shRNA2 to MDA-468; ** P < 0.01, 1.0 mM MMS XRCC1 shRNA1 to MDA-468; *** P < 0.001, 1.0 mM MMS XRCC1 shRNA2 to MDA-468) B) IC_50_ values for MMS in MDA-468 (1.84 ± 0.10 mM) (mean ± SEM), MDA-468 XRCC1 shRNA1 (1.15 ± 0.11), and MDA-468 XRCC1 shRNA2 (1.06 ± 0.07).
(TIF)
