# Supplementary figures and images for: Defective base excision repair in the response to DNA damaging agents in triple negative breast cancer
Source: PLoS One. 2019 Oct 9;14(10):e0223725. doi: 10.1371/journal.pone.0223725 (PMC6785058; doi:10.1371/journal.pone.0223725)

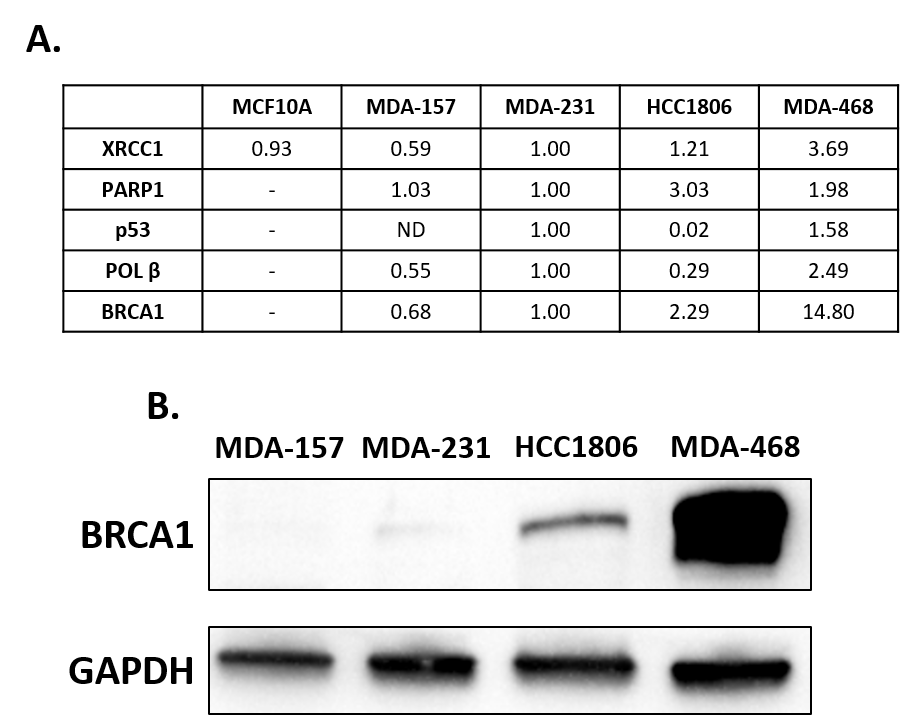

Supplement: S1 Fig — A) Immunoblots from Fig 1A, Fig 1B, and S1B Fig were quantified, normalized to loading control, and reported as fold change relative to MDA-231. ND = no signal detected. B) Lysates for MDA-157, MDA-231, HCC1806, and MDA-468 were probed for BRCA1, with GAPDH serving as a loading control. (TIF) [file pone.0223725.s001.tif]

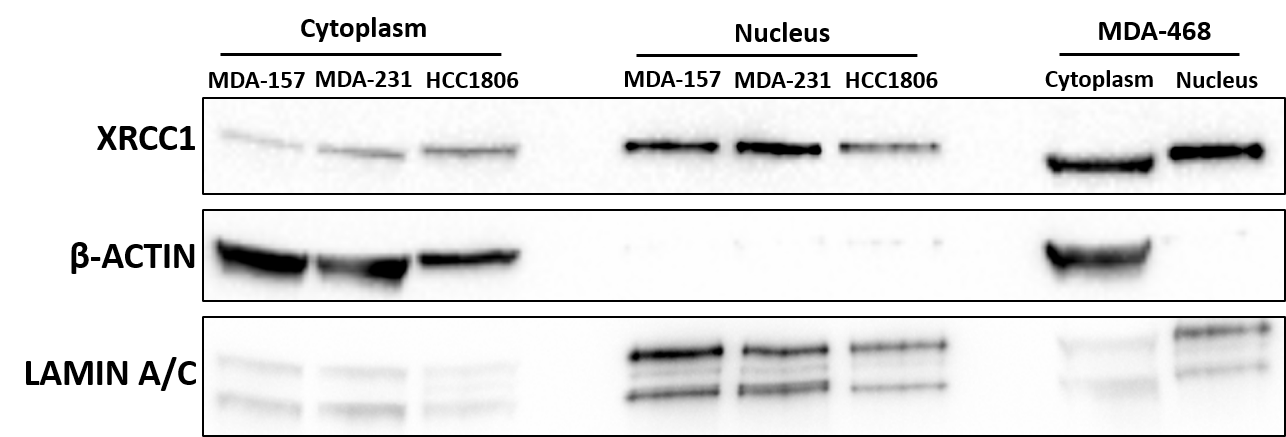

Supplement: S2 Fig — Cytoplasmic and nuclear fractions of all cells were probed for XRCC1. β-ACTIN was used as a loading control for the cytoplasmic fraction while LAMIN A/C was used as a loading control for the nuclear fraction. (TIF) [file pone.0223725.s002.tif]

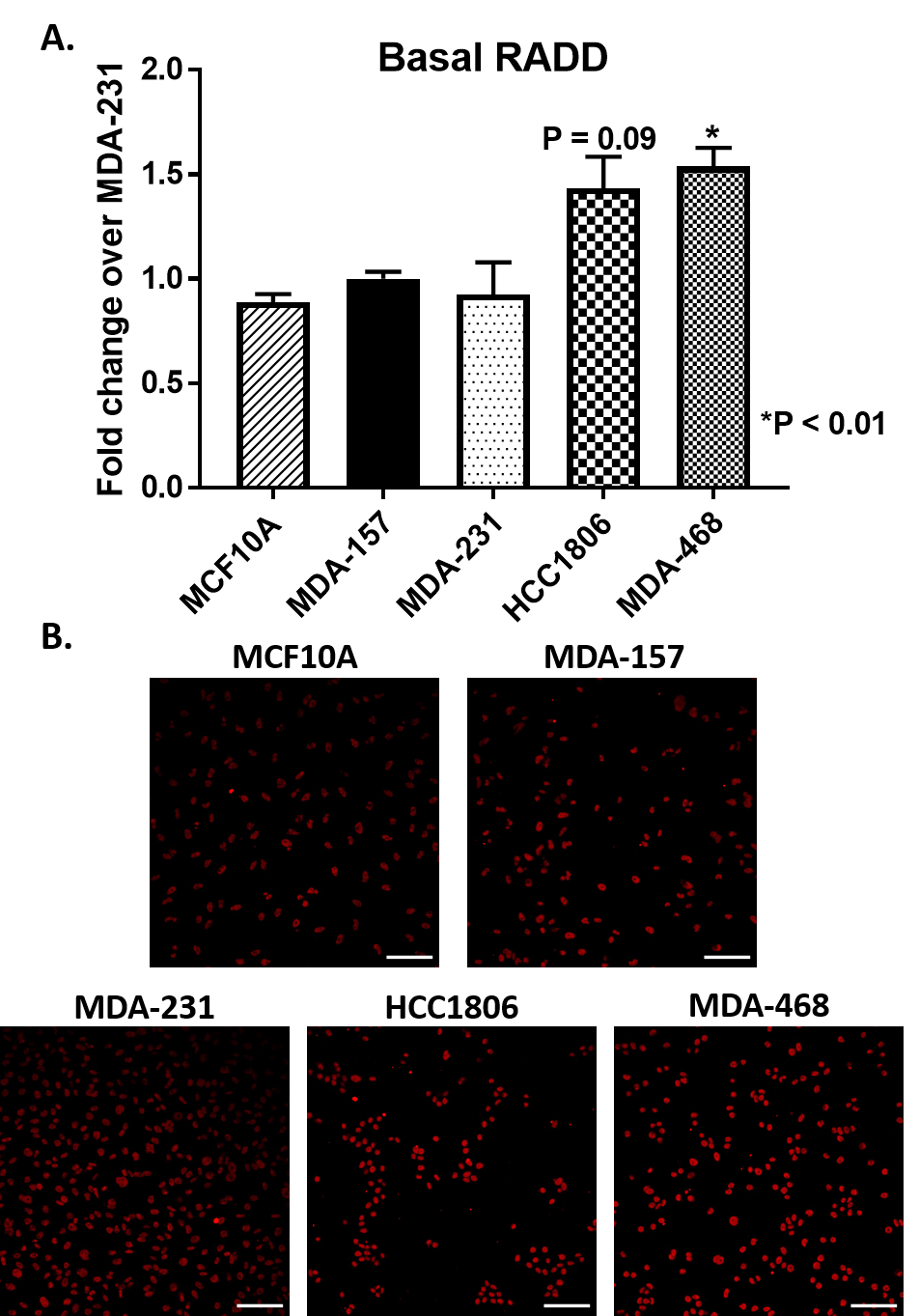

Supplement: S3 Fig — A) Replotting data from Fig 5B in the presence of MCF10A shows similar levels of DNA damage to that of MDA-157 and MDA-231. (P = 0.09, HCC1806 to MDA-231; * P < 0.01, MDA-468 to MDA-231) B) Representative images of basal levels of DNA damage as measure by RADD including MCF10A. Scale bar = 100 μm. (TIF) [file pone.0223725.s003.tif]

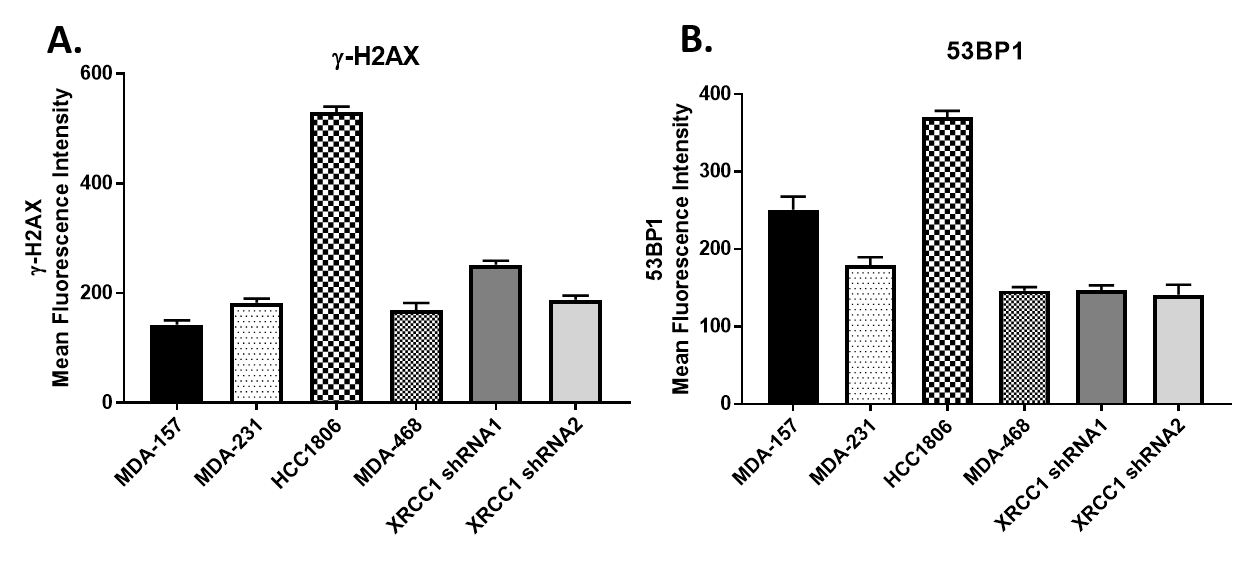

Supplement: S4 Fig — This data indicates that strand breaks are not significantly different in MDA-468 cell lines compared to MDA-468 XRCC1 shRNA cell lines further confirming the ability of RADD to detect broad spectrum DNA damage. (TIF) [file pone.0223725.s004.tif]

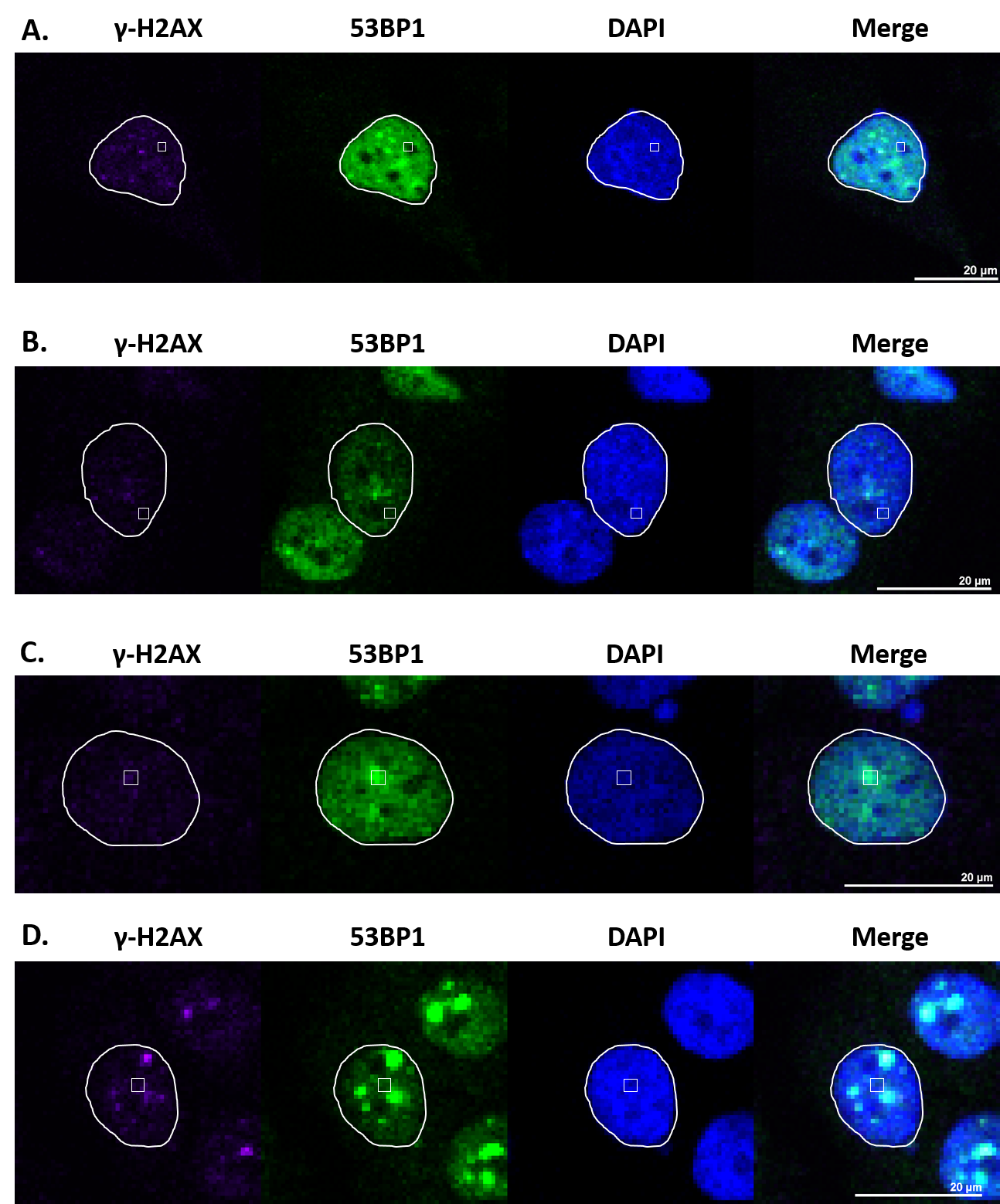

Supplement: S5 Fig — DSB markers 53BP1 (Green) and γ-H2AX (Violet) were stained by immunofluorescence at 10 min after micro-irradiation and representative images are shown for A) MDA-157, B) MDA-231, C) HCC1806, and D) MDA-468. Scale bar = 20 μm. (TIF) [file pone.0223725.s005.tif]

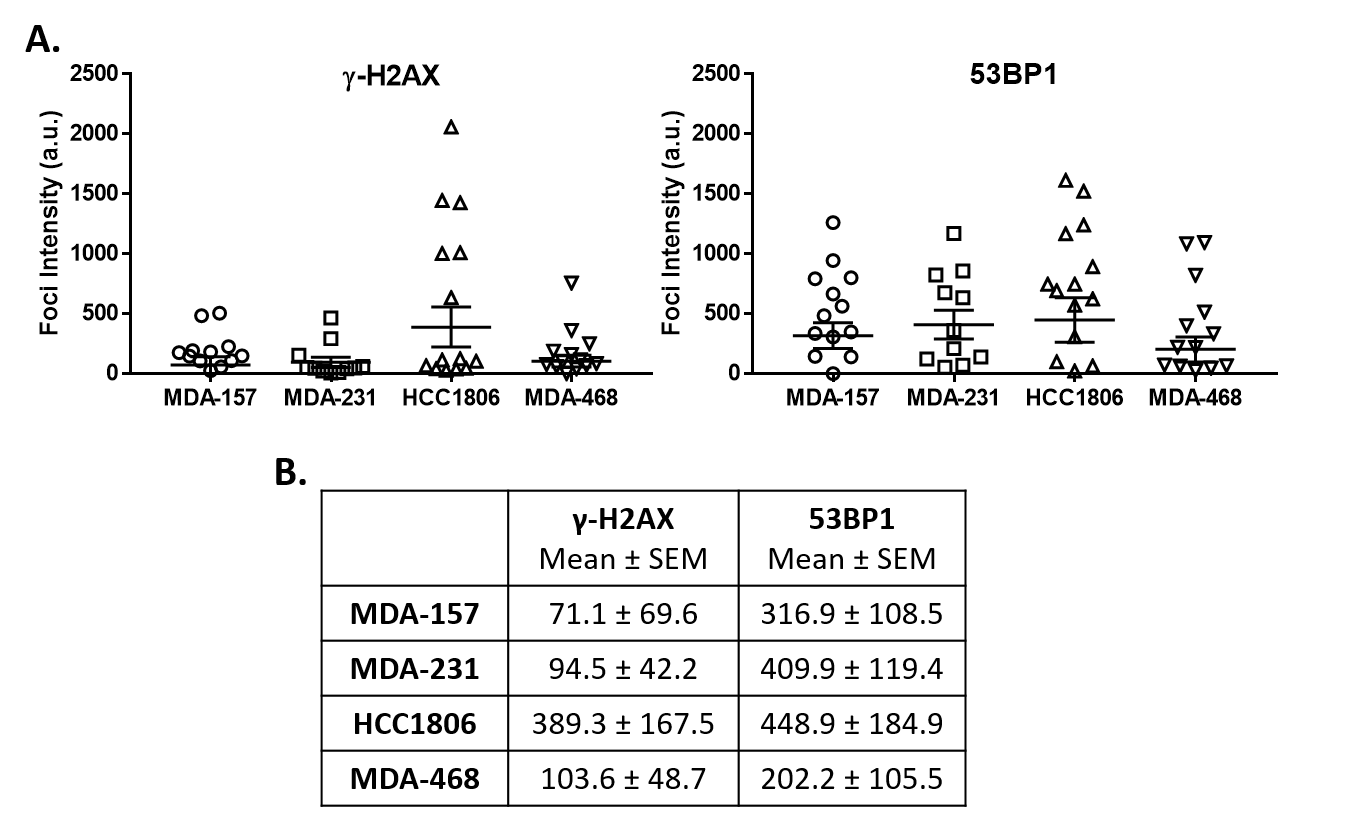

Supplement: S6 Fig — A) Foci Intensity for γ-H2AX (Left), and 53BP1 (Right) for MDA-157, MDA-231, HCC1806, and MDA-468. B) Mean ± SEM for γ-H2AX and 53BP1 from S5A Fig. (TIF) [file pone.0223725.s006.tif]

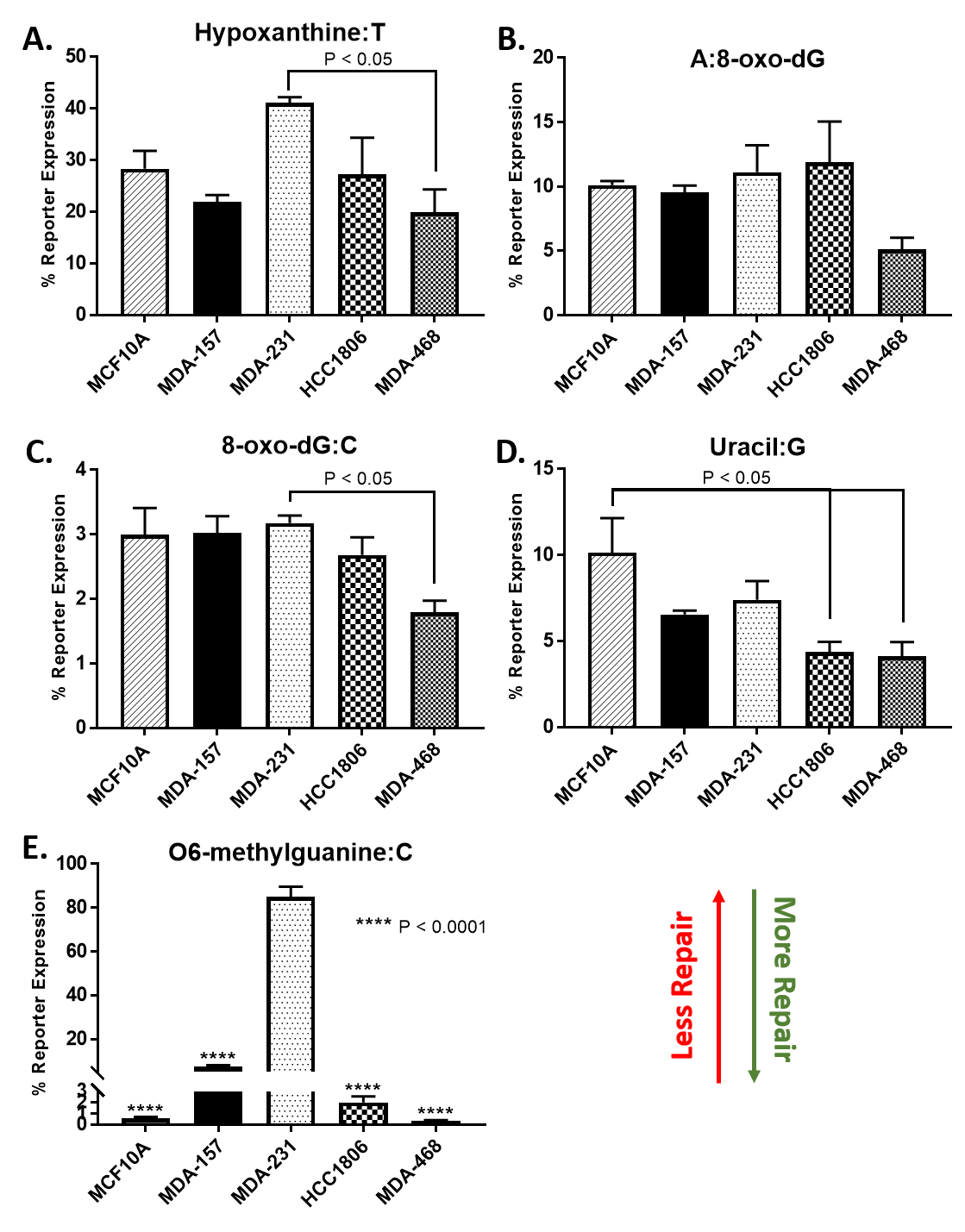

Supplement: S7 Fig — A) Hypoxanthine:T (P < 0.05, MDA-231 to MDA-468), B) A:8-oxo-dG, C) 8-oxo-dG:C (P < 0.05, MDA-231 to MDA-468), D) Uracil:G (P < 0.05, MCF10A to HCC1806, MCF10A to MDA-468), E) O6-methylguanine:C (**** P < 0.0001, MCF10A to MDA-231, MDA-157 to MDA-231, HCC1806 to MDA-231, MDA-468 to HCC1806), as well as an undamaged plasmid to normalize for transfection efficiency. DNA repair capacity is inversely proportional to % reporter expression. (TIF) [file pone.0223725.s007.tif]

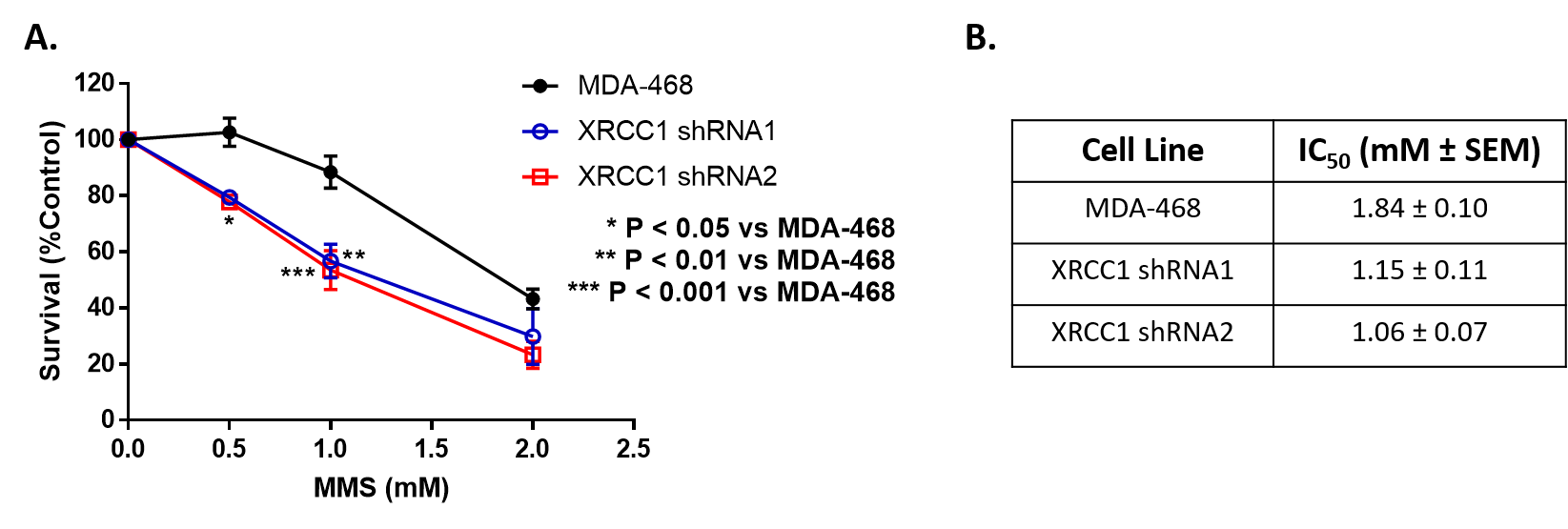

Supplement: S8 Fig — A) MMS sensitivity graphs for MDA-468, MDA-468 XRCC1 shRNA1, and MDA-468 XRCC1 shRNA2. XRCC1 shRNA2 showed significantly more cell death at 0.5 mM MMS compared to MDA-468, while at 1.0 mM MMS both XRCC1 shRNA1 and XRCC1 shRNA2 showed significantly more cell death compared to MDA-468. (* P < 0.05, 0.5 mM MMS XRCC1 shRNA2 to MDA-468; ** P < 0.01, 1.0 mM MMS XRCC1 shRNA1 to MDA-468; *** P < 0.001, 1.0 mM MMS XRCC1 shRNA2 to MDA-468) B) IC50 values for MMS in MDA-468 (1.84 ± 0.10 mM) (mean ± SEM), MDA-468 XRCC1 shRNA1 (1.15 ± 0.11), and MDA-468 XRCC1 shRNA2 (1.06 ± 0.07). (TIF) [file pone.0223725.s008.tif]
